# The Original Panelites: An Indignant Protest

**Published:** 1913-01-25

**Authors:** 


### The Editor's Letter-Box.

### The Original Panelites: An Indignant Protest.

*To the Editor of* The Hospital.

Sir,—I have heard some of my friends express sympathy with the doctors who appear to have but small regard for either truth or honesty. I should like to protest against any sympathy being offered to the so-called panelites, who have betrayed their fellow-workers and broken their pledges.

At school I was taught that "An Englishman's word was his honour." Some of a body of, I suppose, the best educated professional body of men in the world, have disgraced this time-honoured and respected quotation, but I am glad to say a few have repented, and attempted to make a small reparation by resigning. Honest men respect them.

Far back in history I have read of a man who, without signing a pledge over a sixpenny stamp or otherwise, broke his promise to his colleagues in a moment of temptation, afterwards he was so ashamed of himself that he refused to make any use of his ill-gotten reward, but committed suicide, having realised the enormity of the crime of having broken his promise. He was not an Englishman, well educated, with a good postal address and a telephone number, like many panelites, but a man whom (having done his best to purge himself of his sin) many honest men would rather meet than one of the panelites who say they are not ashamed of their disgraceful and cowardly behaviour. I am of course mentioning one "Judas Iscariot," who went out and hanged himself.

If only all the panel doctors would either resign or "Go now and do likewise" it would do a great deal towards helping the public to again consider the medical profession an honourable, honest, and conscientious one.—Yours faithfully, C. E. A. H.

Gloucester Terrace, W., January 21.

### Service in Hospital Wards.

*To the Editor of* The Hospital.

